# Effect of Dietary Intervention Designed with Behavior Change Wheel on Compliance with Dietary Control in Women with Gestational Diabetes Mellitus: Study Protocol for a Randomized Controlled Trial

**DOI:** 10.3390/ijerph191710726

**Published:** 2022-08-28

**Authors:** Jingqi Xu, Yuanyuan Wu, Zhijie Zou, Xiaoli Chen

**Affiliations:** 1 School of Nursing, Wuhan University, Wuhan 430072, China; 2 Suizhou Hospital, Hubei University of Medicine (Suizhou Central Hospital), Suizhou 441300, China

**Keywords:** behavior change wheel, dietary control, gestational diabetes mellitus, food exchange serving, glycemic load

## Abstract

Background and Purpose: Previous studies have shown that women with GDM can benefit from following dietary recommendations, which are based on food exchange serving (FES) and glycemic load (GL), but compliance with dietary recommendations in women with GDM is not ideal. Therefore, the aim of this paper is to design a dietary intervention program based on behavior change wheel (BCW) to affect GDM women’s compliance with FES based on GL, and to compare the effects of this dietary intervention program versus general dietary management on compliance with dietary recommendations, improving maternal glucose metabolism, and reducing adverse pregnancy outcomes in women with GDM. Methods: This paper is a methodological description of a two-arm randomized controlled trial. In this study, eligible women with GDM will be recruited and divided into the control group (*n* = 30) and the intervention group (*n* = 30). Women with GDM will respectively receive general dietary management (control group) and dietary intervention designed with BCW (intervention group) until after delivery. Information about pregnant women will be collected through questionnaires or prenatal and delivery records. Conclusion: This randomized controlled trial is designed specifically for women with GDM to achieve effective blood glucose control by strengthening GDM women’s compliance with dietary recommendations. If this dietary intervention designed with BCW proves to be effective, then BCW may deserve to be applied to more areas of self-management in women with GDM.

## 1. Introduction

Gestational diabetes mellitus (GDM) has become a new global public health problem. According to the latest data from the International Diabetes Federation (IDF), nearly 17 million pregnant women worldwide currently suffer from GDM [1]. With obesity, advanced gestational age, and sedentary lifestyles becoming more common, the number of women with GDM will continue to increase [2,3]. It is well known that GDM has both long-term and short-term adverse effects on the health of pregnant women and their offspring. These adverse effects include pre-eclampsia, cesarean delivery, and later development of type 2 diabetes in women with GDM [4,5], as well as macrosomia, perinatal mortality, and higher living risk of obesity and type 2 diabetes in offspring born to mothers with GDM [4,6]. To avoid the consequences of GDM, achieving and maintaining euglycemia become the key to care for women with GDM [7].

According to the recommendations of the American Diabetes Association (ADA), the first choice for women with GDM to achieve glycemic targets is lifestyle and behavioral management, which includes medical nutrition therapy (MNT), physical activity, and weight management [8]. Among them, MNT is the most basic link in diabetes treatment [9]. The appropriate nutritional therapy for GDM should aim to ensure the intake of high-quality ingredients to meet healthy pregnancies while achieving euglycemia [10]. However, striking the right balance between the two objectives is a challenge due to the traditional Chinese belief that pregnant women should eat more [11].

The traditional food exchange serving (FES) is a classic meal planning of MNT [12], which is designed according to dietary recommendations. By following the traditional FES, women with GDM can control daily caloric intake and the proportion of three thermogenic nutrients to just enough to meet the needs for healthy pregnancy [12]. Currently, the traditional FES has been widely used in dietary intervention for diabetic patients in East Asian countries [13,14,15], and its effectiveness has been proven [13,16]. However, despite such advantages of the traditional FES, it cannot distinguish differences in glycemic response caused by various foods within the same food type [13]. This leads to the possibility that people with diabetes may still choose foods that have a relatively large impact on blood glucose, although following the traditional FES. Therefore, FES based on glycemic load (GL) was put forward as a new meal planning of MNT [17].

GL was proposed by Salmeron et al. in 1997, which is used to reflect the increasing potential of insulin demand caused by carbohydrates in foods [18]. In 2006, according to dietary habits of Chinese people, Sun et al. developed the new food exchange system based on GL [17]. Different from the traditional FES, FES based on GL suggests that people with diabetes should refer to GL value of each food before selecting foods in the last step of the traditional FES [17]. Existing evidence shows that following FES based on GL could help women with GDM to achieve better results in controlling blood glucose compared to following the traditional FES [19,20]. A meta-analysis of 29 studies also showed that ethnic Chinese women with GDM can benefit from choosing foods with low GL value to improve glucose metabolism and reduce the risks of adverse pregnancy outcomes [21].

The benefits of following dietary recommendations for women with GDM are evident, but whether they actually benefit from it depends on their compliance with dietary recommendations. Mustafa et al. found that only 31.2% of 313 women with GDM in New Zealand had high compliance with dietary commendations by analyzing the matching degree between maternal dietary intake and dietary recommendations [22]. A cohort study conducted in Denmark revealed that only 35% of 336 women with GDM who received MNT were highly compliant with dietary guidelines [23]. In addition, Guo Meiying et al. found that only 46.7% of 139 Chinese women with GDM used the traditional FES to prepare food and control energy intake [24]. Due to these unsatisfactory conditions, it is necessary to design a dietary intervention program that can optimize existing dietary management so as to improve GDM women’s compliance with dietary recommendations.

**Figure 1:**
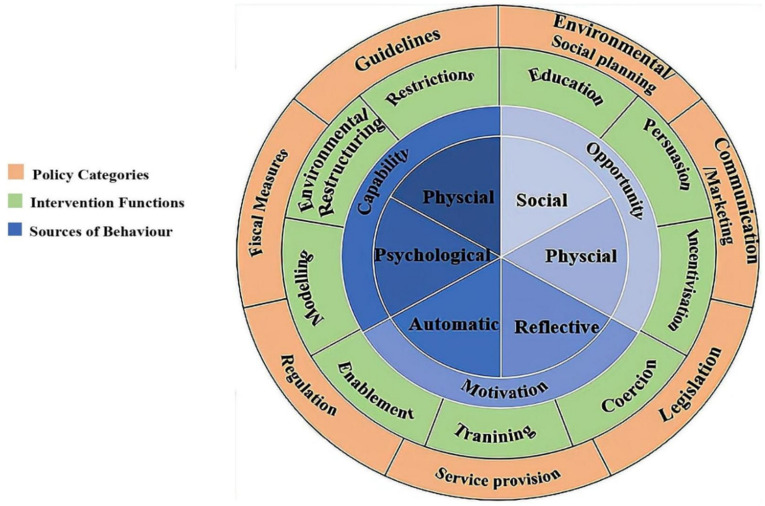
Behavior Change Wheel (adapted from Michie et al. [25]).

The Behavior Change Wheel (BCW) is a framework for developing intervention, which is formed by the integration of 19 theoretical frameworks related to behavioral change [25]. BCW has three layers (see Figure 1). Its core is the Capability-Opportunity-Motivation-Behavior system (COM-B system). Michie et al. proposed that capability, opportunity, and motivation are essential conditions to promote the occurrence or change of behaviors [25]. Furthermore, nine intervention functions and seven policy categories are wrapped by layer on the outside of the core [25]. Based on BCW, intervention designers can identify defects in one or more of the three essential conditions in the COM-B system, then effectively select the most appropriate intervention functions that can correct the defects and available policies. Currently, BCW has been used in several health fields, such as community health promotion, health management, and nursing [26,27]. However, BCW has not been used to improve compliance with dietary recommendations among women with GDM.

### Propose

The purposes of this study are to design an intervention program based on BCW to affect GDM women’s compliance with FES based on GL and to compare the effects of this dietary intervention program versus general dietary management on compliance with dietary recommendations, improving maternal glucose metabolism and reducing adverse pregnancy outcomes in women with GDM. This paper is a methodological description of the intervention design and trial protocol.

## 2. Research Hypothesis

We hypothesize that after the intervention, women with GDM who receive the intervention program designed based on BCW will have (1) higher scores of self-management behavior and self-efficacy, (2) better results of maternal glucose metabolism, and (3) lower incidences of common adverse pregnancy outcomes than women with GDM who receive general dietary management.

## 3. Methods

### 3.1. Trial Design and Ethical Approvals

This is a two-arm randomized controlled trial study with parallel designs. The duration of the intervention is expected to take about 6 months. The trial study has been approved by Medical Ethics Committee at Wuhan University School of Medicine (No. 2021YF0048). It was registered by ClinicalTrials.gov on 18 April 2022. The registration number is ChiCTR2200058872.

### 3.2. Research Setting and Participant Eligibility Criteria

The study will be conducted in the obstetric clinic of a Grade 3A hospital in Wuhan, Hubei Province. Pregnant women diagnosed with GDM by a clinical 75 g oral glucose tolerance test (OGTT) at 24 weeks of gestation are eligible to participate. The glucose concentration thresholds of pregnant women accord with IADPSG criteria in 2010 [28]: Fasting blood glucose (FBG) ≥ 5.1 mmol/L and/or 1-h plasma glucose after taking sugar ≥ 10.0 mmol/L and/or 2-h plasma glucose after taking sugar ≥ 8.5 mmol/L. Furthermore, women with GDM who are eligible must meet the following inclusion criteria: (1) Aged between 18 and 40 years, (2) natural pregnancy, (3) singleton pregnancy verified by ultrasound, (4) have adequate cognitive and behavioral abilities, (5) no previous endocrine-related diseases, such as hyperthyroidism, hypothyroidism, and Cushing’s syndrome, (6) voluntarily participate in this study and sign the informed consent form.

### 3.3. Participant Exclusion Criteria

Pregnant women who meet the following criteria will be excluded from the study: (1) Those who have pre-pregnancy diabetes mellitus, (2) ultrasound results showed that the fetus had serious congenital abnormalities, (3) pre-existing pregnancy comorbidities, such as mental illness, heart failure, renal failure, and severe liver disease, (4) existing pregnancy complications, such as severe anemia, threatened abortion and fetal intrauterine growth restriction, (5) using drugs that may affect OGTT results, such as steroids or immunosuppressants, (6) those with a history of smoking, alcohol abuse, or drug abuse, (7) those who have special dietary requirements, such as vegans or those who have special food allergies.

### 3.4. Participant Shedding Criteria

In the following cases, participants will be withdrawn if: (1) Pregnant women who opt to terminate their pregnancy during the study period, (2) pregnant women who do not wish to continue to participate in the study, (3) pregnant women who have difficulties in continuing to participate in this study due to the following physical conditions that happen during the intervention process, such as vaginal bleeding, threatened premature delivery, placental abnormalities.

### 3.5. Sample Size

Due to the lack of prior data about applying BCW in dietary intervention in women with GDM, a pilot study will be carried out to provide a comprehensive estimate of the effect size of the intervention. The recruitment goal of the pilot study is 30 pregnant women with GDM for each group [29]. No a priori hypotheses of effect size to identify the ineffectiveness of a comprehensive study were proposed since we considered that the available data were insufficient to create these meaningfully. The minimum sample size for the future study will be calculated based on the results of the pilot study.

### 3.6. Recruitment

Participants will be recruited when pregnant women return to the obstetrics clinic for their 75 g OGTT results. Obstetricians will explain the purpose and significance of this study and emphasize that the trial will be conducted in strict confidentiality. Women with GDM who volunteer to participate in this study will be enrolled immediately after signing the informed consent forms.

### 3.7. Randomization and Blinding

Researchers will use the study website (www.random.org/lists/, (accessed on 19 May 2022)) to create random numbers that correlate to the serial numbers before recruiting participants. The ratio of random allocation is 1:1. Eligible participants will be sequentially recruited and assigned the predetermined random number. Participants with a random number of 1–30 will be divided into the dietary intervention group based on BCW (intervention group), while those with a random number of 31–60 will be divided into the general dietary management group (control group). In this study, participants and interveners will inevitably know the content of the intervention. Therefore, researchers will only ensure the separation of interveners and result assessors.

### 3.8. Randomized controlled trial (RCT) Design

At enrollment, all participants will receive paper guidance materials for dietary control, including a guidebook and the food exchange table. The guidebook records the methods for calculating pre-pregnancy BMI and daily energy requirements for women with GDM (see Supplementary Files S1 and S2), as well as how to convert daily energy requirements into the number of food exchange servings (see Supplementary File S2). The food exchange table shows the GL value and the raw weight of an exchange serving of a certain food. Pregnant women could follow the distribution principles of three major nutrients (daily intake of carbohydrates, protein, and fat account for 50%, 60%, 15%, 20%, and 25–30% of the total energy, respectively. The daily intake of carbohydrate should not be less than 150 g) and the principle of “small and frequent meals” (breakfast, lunch, and dinner + extra 2–4 meals) indicated in the guidebook, and combined with guidance materials to design daily recipes.

#### 3.8.1. Control Group

Pregnant women in the control group will receive general dietary management, including an individual primary consultation at enrollment, two additional consultations at reviews, and online consultations [30,31]. Online consultations will be conducted at three days after enrollment and at 7:00 p.m. every Thursday.

In individual primary consultation, researchers will explain the importance of dietary control, the principles of dietary control, and how to design daily recipes according to FES based on GL to pregnant women in the control group. Researchers will also explain the content of the paper guidance materials to help pregnant women understand the distribution principles of three major nutrients and how to follow the principle of “small and frequent meals”.

In the online consultation at three days after enrollment, researchers will evaluate the dietary behaviors of pregnant women in the past three days and correct the wrong dietary behaviors. In the online consultation at 7:00 p.m. every Thursday, researchers will provide an online guidance team composed of an obstetrician, a dietitian, and two postgraduate nursing students to answer pregnant women’s questions and conduct health education in the WeChat communication group.

In the first addition consultation at the first week after enrollment, researchers will decide on the next phase of dietary management for pregnant women according to the results of fasting blood glucose (FBG) and 2 h postprandial blood glucose (2 h PBG). For pregnant women who need to use insulin, after the clinician has specified the insulin dosage, researchers will adjust pregnant women’s dietary management options in conjunction with the clinician’s advice. For pregnant women who need to make changes to their dietary control, researchers will review pregnant women’s eating behaviors over the past week and correct wrong eating behaviors. For pregnant women who need to stick to the original plan, researchers will not give other guidance.

In the second additional consultation at 28 weeks of gestation, researchers will instruct pregnant women to increase 450 kcal on their daily basal energy requirements calculated from their pre-pregnancy BMI and body energy coefficient [32], so as to properly adjust exchange servings of a certain food.

#### 3.8.2. Intervention Group

Pregnant women in the intervention group will receive dietary intervention designed based on BCW, including an individual primary consultation at enrollment, two additional consultations at reviews, and online consultations. Each essential condition in COM-B system often corresponds to multiple intervention functions, policies, and behavioral change techniques (BCTs). Therefore, researchers will select intervention components according to APEASE criteria (affordable, practical, efficacious, acceptable, safe, and equitable) in this study [25]. The matrix of links between the components of BCW (COM-B system, intervention functions, and policy category) for the intervention is described in Table 1.

In the individual primary consultation at enrollment, four intervention strategies will be implemented: (1) Determining the positive and adverse aspects of pregnant women’s current dietary behaviors, and emphasizing the benefits of positive behaviors and the consequences of adverse behaviors, (2) clarifying the importance and principles of dietary control for the treatment of GDM and helping pregnant women to set goals for glycemic control, (3) providing the same guidance materials and explaining the content of the paper guidance materials as the control group for designing daily recipes, showing the additional food atlas showing the shape, volume ration and energy value of food under different grams, different dry and wet states and different cooking methods, and providing the same type of simple food scales to pregnant women for determining the amount of food intake, training pregnant women to design the daily recipes.

In the online consultation at three days after enrollment, three intervention strategies will be implemented: (1) Evaluating the dietary behaviors of pregnant women in the past three days and correcting the wrong dietary behaviors as the control group, (2) informing pregnant women’s main caregivers about the importance of dietary control for women with GDM and the benefits of providing good family support, i.e., enhance pregnant women’s motivation to comply with dietary control, (3) putting forward some suggestions on how to control the diet when participating in a multi-person dinner. For example, use a spoon to estimate the intake amount of a certain food. In the online consultation at 7:00 pm every Friday, two intervention strategies will be implemented: (1) Providing the same online guidance team to answer pregnant women’s questions and conduct health education in the WeChat communication group as the control group, (2) helping pregnant women to design recipes for the next week’s diet.

In the first additional consultation in one week after enrollment, two intervention strategies will be implemented: (1) Deciding the next phase of treatment for pregnant women according to the reexamination results of maternal glucose metabolism, just as researchers do for the control group, (2) setting up the collaborative dietary management group of three women with GDM and encourage them to share dietary control experience, so as to achieve peer education and model setting.

In the second additional consultation at 28 weeks of gestation, researchers will instruct pregnant women to increase 450 kcal on their daily basal energy requirements calculated from their pre-pregnancy BMI and body energy coefficient [32], so as to properly adjust exchange servings of a certain food.

## 4. Measures

Primary outcomes for compliance with dietary recommendations include self-management behaviors and self-efficacy of dietary control, and the average daily GL value. Secondary outcomes for maternal glucose metabolism include results of OGTT, FBG, 2 h PBG, and HbA1c. Secondary outcomes for adverse pregnancy outcomes include non-normal gestational weight gain (GWG) (excessive GWG or insufficient GWG), insulin use during pregnancy, cesarean section, premature delivery, macrosomia, and fetal intrauterine growth restriction. The timeliness of data collection is shown in Table 2.

### 4.1. Body Measurement

The height and weight of pregnant women will be measured to calculate pre-pregnancy BMI and gestational weight gain. Pregnant women’s height and weight will be measured via a height and weight measuring instrument, and the results will be accurate to one decimal place.

### 4.2. Baseline Characteristics Measurement

The baseline characteristics of pregnant women will be measured through the self-designed demographic and pregnancy-related questionnaires, including gestational age, occupation, education level, income, residence place, previous history of disease, gestational weeks, fetal number, existing pregnancy complications, previous pregnancy comorbidities, history of smoking, alcohol abuse and drug abuse, and information about special diet.

### 4.3. Assessment of Compliance with Dietary Recommendations

The self-management behaviors of dietary control will be assessed by a questionnaire designed by researchers based on literature review and dietary recommendations, which includes the following questions: “How many days a week do you eat according to the principle of “breakfast, lunch, and dinner, and 2–4 extra meals?”; “How many days a week do you match the staple food according to the principle that the ratio of coarse grain to fine grain is 1:2 or 1:4?”; “How many days a week do you subtract the corresponding staple food when eating starchy foods (for example, 100 g of potatoes can be exchanged with 25 g of rice, millet, and sorghum rice)?”; “How many days a week do you follow the food exchange strategy as recommended by the medical staff?”; “How many days a week do you refer to the GL value of food when choosing foods?” Each item is scored according to the number of days in which behaviors occurred, with 0 to 7 points assigned from 0 to 7 days. The total score ranges from 0 to 35 points. 0–11 points, 12–23 points, and 24–35 points indicate low, medium, and high levels of dietary self-management behavior, respectively.

The self-efficacy of participants will be assessed by the Chinese version of the Diabetes Management Self-Efficacy Scale (C-DMSES). The DMSES was originally developed by van der Bijl et al. [33]. Wu et al. have translated it into Chinese version [34]. In this study, we will use the nutrition-related subscale of C-DMSES to assess the self-efficacy of dietary control in women with GDM. The nutrition-related subscale has nine items, and each is rated on a 5-point scale (1 = “strongly disagree” 5 = “strongly agree”) [34]. Higher scores indicate better self-efficacy. The Cronbach’s alpha of the nutrition-related subscale of C-DMSES is 0.93 [34].

Maternal dietary intake will be recorded through the 24-h dietary recalls questionnaire. Participants will be asked to record dietary information for two business days and one holiday in one week and record the types and amounts of food intake, including main meals, extra meals, and snacks. In addition, the average daily GL value will be taken as the mean of the total GL value of all food exchange servings included in the dietary records. Food exchange servings are converted from the total energy of food consumed.

### 4.4. Maternal Glucose Metabolism Measurement

The laboratory department of the hospital will be responsible for measuring the experimental indicators related to maternal glucose metabolism. The experimental indicators include OGTT, FBG, 2 h PBG, and HbA1c, which will be measured at the study entrance and again at 37 weeks of gestation. Data on the results of these experimental indicators will be collected from the prenatal and delivery records of participants.

### 4.5. Pregnancy Outcomes

Information about adverse pregnancy outcomes will be collected from prenatal and delivery records, including excessive GWG or insufficient GWG, insulin use during pregnancy, cesarean section, premature delivery, macrosomia, and fetal growth restriction.

## 5. Data Collection

Data will be collected by a specially-assigned person who is unaware of participant allocation and intervention content.

## 6. Statistical Analysis

All quantitative analysis will be conducted through IBM SPSS Statistics 24.0 software (IBM Corp. Released 2016. IBM SPSS Statistics for Windows, Version 24.0., IBM Corp.: Armonk, NY, USA). The baseline characteristics of participants will be presented by descriptive statistics. The continuous variables will be described by means and standard deviation, or medians and quartiles. The frequencies (N) and percentages (%) will be used for categorical variables. The analysis of variance (ANOVA), Kruskal–Wallis, test or Chi-square test will be used to describe the mean differences of continuous variables and categorical variables between the two groups at baseline. The explored assessment will be analyzed by repeated measures multivariate analysis of variance (MANOVA), and then the post-hoc pairwise comparison will be made. Effect sizes were calculated with Cohen’s d. P value of 0.05 will be considered the significant level for all outcomes.

## 7. Discussion

This randomized controlled trial is designed specifically for women with GDM to achieve effective blood glucose control by strengthening GDM women’s compliance with dietary recommendations. To our knowledge, this is the first study to use the BCW model as the theoretical framework for dietary intervention in women with GDM. Compared with other nutritional interventions for GDM women, this study identifies one or more effective intervention functions of three essential conditions in COM-B system and links interventions to BCTs, so as to avoid the omission of important interventions. Furthermore, the impact of environmental factors on behavior was considered in this study. For example, the intervention object is not limited to women with GDM, but also includes pregnant women’s main caregivers. The capability and motivation of primary caregivers to assist women with GDM in managing their diet can also be improved through the intervention. Women with GDM can get more family support which will create more opportunities for women with GDM to control their diet according to dietary recommendations so as to achieve better results in controlling blood glucose. Women with GDM can receive more family support, which will create more opportunities for women with GDM to comply with dietary recommendations, thus achieving better glycemic control.

It is worth noting that the study still has some limitations. First, dietary management behaviors and self-efficacy of women with GDM are evaluated through several self-reported questionnaires, which may cause reporting bias. Second, a questionnaire measure used in this study is not validated. Further testing in a larger sample of patients with GDM is needed. Third, researchers will be involved throughout the study, which may lead to the subjective bias of the subjects or personnel, thus affecting study results.

## 8. Conclusions

Nevertheless, it remains exciting if this intervention program designed with BCW proves effective in improving compliance with dietary recommendations and maternal glucose metabolism and reducing adverse pregnancy outcomes. The success of this intervention will provide medical professionals with a novel intervention design idea that BCW can also be applied to improve other self-management behaviors in women with GDM, such as improving compliance with glucose self-monitoring and physical activity. This may provide a boost to the clinical management of GDM.

## Figures and Tables

**Table 1:** Matrix of links between the components of the BCW model (COM-B model, intervention functions, and policy category) for the intervention.

Mode of Delivery	Time	Intervention Subjects	Policy Category	Intervention Functions: Description of Intervention Strategies	Capability		Opportunity		Motivation	
					Phys	Psych	Social	Phys	Refl	Aut
Individual primarily consultation	At enrollment	Pregnant women	Communication; Guideline;	*Persuasion*: Identify the positive and negative aspects of current dietary behaviors, and inform about the benefits of adjusting dietary behaviors;					√	√
			Communication; Guideline;	*Education*: Set goals of blood glucose control and clarify the importance and principles of dietary management for GDM, Explain the content of paper guidance materials,		√			√	
			Environmental planning	*Environment restructuring*: Provide paper guidance materials for designing daily recipes. Show the food atlas, and provide sample food scales to pregnant women for determining the amount of food intake;			√	√		√
			Communication; Guideline;	*Training*: Guide pregnant women in designing daily recipes	√	√		√		√
Online consultation	At three days after enrollment	Pregnant women	Communication;	*Incentivisation and enablement*: Evaluate the dietary behaviors of pregnant women in the past three days and correct the wrong dietary behaviors;	√	√	√	√	√	√
		Pregnant women’s main caregivers	Communication; Guideline;	*Education, persuasion and environment restructuring*: Inform the main caregivers about the importance of dietary management for women with GDM and the benefits of providing good family support that can enhance the motivation of pregnant women to adhere to MNT;		√	√	√	√	√
		Pregnant women	Communication; Guideline;	*Persuasion and enablement*: Put forward some suggestions on how to manage the diet when eating out or eating at home;	√	√	√	√	√	√
	At 7:00 pm every Friday	Pregnant women	Service provision; Communication;	*Enablement*: Provide the online guidance team to answer pregnant women’s questions and conduct health education as the control group, and help pregnant women design recipes for the next week’s diet.	√	√	√	√	√	√
The first additional consultation at review	At one week after enrollment	Pregnant women	Communication; Guideline; Service provision;	*Persuasion, incentivization, and enablement*: Re-measure blood glucose, and look back and analyze the dietary-related reasons for the positive or reverse results of controlling blood glucose	√	√	√	√	√	√
			Communication;	*Education, persuasion, and modeling*: Set up the three-person cooperation group to achieve peer education		√	√		√	√
The second additional consultation at review	At 28 weeks of gestation	Pregnant women	Communication;	*Education*: Recalculate total daily energy requirements, and adjust exchange servings of a certain food for meeting the need for the healthy pregnancy		√			√	

**Table 2:** Timeliness of Data Collection.

	Enrollment	A Week after Enrollment	28 Weeks of Gestation	37 Weeks of Gestation	After Intervention (Before Delivery)	After Delivery
**Baseline Characteristics**						
Demographic characteristics	√					
Pregnancy-related characteristics	√					
**Body Characteristics**						
Weight	√				√	
Height	√					
**Maternal Glucose Metabolism**						
75 g OGTT	√					
FBG		√		√		
2 h PBG		√		√		
HbA1c		√		√		
**Compliance with Dietary recommendations**						
Self-management behavior				√		
Self-efficacy				√		
Average daily GL value			√	√		

Abbreviations: 75 g OGTT, 75 g oral glucose tolerance test; FBG, fasting blood glucose ; 2 h BG, 2 h postprandial blood glucose; GL, glycemic load.

## Data Availability

At the end of the study, the data used to support the results of this study can be obtained from the corresponding authors according to the requirements. This study does not involve in participants’ individual data.

## Supplementary Materials

The following are available online at https://www.mdpi.com/article/10.3390/ijerph191710726/s1. Supplementary Files S1–S3.

## References

[1] IDF Diabetes Atlas 2021. https://diabetesatlas.org/atlas/tenth-edition/.

[2] Laine M.K., Kautiainen H., Gissler M., Raina M., Aahos I., Järvinen K., Pennanen P., Eriksson J.G. (2018). Gestational diabetes in primiparous women-impact of age and adiposity: A register-based cohort study. Acta Obstet. Gynecol. Scand.

[3] Juan J., Yang H. (2020). Prevalence, Prevention, and Lifestyle Intervention of Gestational Diabetes Mellitus in China. Int. J. Environ. Res. Public Health.

[4] Plows J.F., Stanley J.L., Baker P.N., Reynolds C.M., Vickers M.H. (2018). The Pathophysiology of Gestational Diabetes Mellitus. Int. J. Mol. Sci..

[5] Tobias D.K., Stuart J.J., Li S., Chavarro J., Rimm E.B., Rich-Edwards J., Hu F.B., Manson J.E., Zhang C. (2017). Association of History of Gestational Diabetes With Long-term Cardiovascular Disease Risk in a Large Prospective Cohort of US Women. JAMA Intern. Med..

[6] Wang J., Wang L., Liu H., Zhang S., Leng J., Li W., Zhang T., Li N., Li W., Baccarelli A.A. (2018). Maternal gestational diabetes and different indicators of childhood obesity: A large study. Endocr. Connect..

[7] Berger H., Gagnon R., Sermer M. (2019). Guideline No. 393-Diabetes in Pregnancy. J. Obstet. Gynaecol. Can..

[8] Draznin B., Aroda V.R., Bakris G., Benson G., Brown F.M., Freeman R., Green J., Huang E., Isaacs D., Kahan S. (2022). 15. Management of Diabetes in Pregnancy: Standards of Medical Care in Diabetes-2022. Diabetes Care.

[9] Reader D.M. (2007). Medical nutrition therapy and lifestyle interventions. Diabetes Care.

[10] Mahajan A., Donovan L.E., Vallee R., Yamamoto J.M. (2019). Evidenced-Based Nutrition for Gestational Diabetes Mellitus. Curr. Diab. Rep..

[11] Wah Y.Y.E., McGill M., Wong J., Ross G.P., Harding A.J., Krass I. (2019). Self-management of gestational diabetes among Chinese migrants: A qualitative study. Women Birth.

[12] Zhu T., Wang X., Zhang Y. (2020). Research on Interaction Design of Diabetes Diet Health Based on GL Food Exchange Serving. J. Phys. Conf. Ser..

[13] Wang H., Bian X., Cheng X., Hua Y. (2016). Impact of different food exchange method on patients with gestational diabetes mellitus. Chin. J. Diabetes.

[14] Kitamura S. (1994). Diet therapy and food exchange lists for diabetic patients. Diabetes Res. Clin. Pract..

[15] Jin S.M., Ahn J., Park J., Hur K.Y., Kim J.H., Lee M.K. (2021). East Asian diet-mimicking diet plan based on the Mediterranean diet and the Dietary Approaches to Stop Hypertension diet in adults with type 2 diabetes: A randomized controlled trial. J. Diabetes Investig..

[16] Lim H.M., Park J.E., Choi Y.J., Huh K.B., Kim W.Y. (2009). Individualized diabetes nutrition education improves compliance with diet prescription. Nutr. Res. Pract..

[17] Sun J., Shen X., Zong M., Chen Y., Feng Y., Chen X. (2006). A novel food exchange serving based on glycemic load in diet therapy for diabetes. Acta Nutr. Sin..

[18] Salmerón J., Ascherio A., Rimm E.B., Colditz G.A., Spiegelman D., Jenkins D.J., Stampfer M.J., Wing A.L., Willett W.C. (1997). Dietary fiber, glycemic load, and risk of NIDDM in men. Diabetes Care.

[19] Lv S., Yu S., Chi R., Wang D. (2019). Effects of nutritional nursing intervention based on glycemic load for patient with gestational diabetes mellitus. Ginekol. Pol..

[20] Ma W.J., Huang Z.H., Huang B.X., Qi B.H., Zhang Y.J., Xiao B.X., Li Y.H., Chen L., Zhu H.L. (2015). Intensive low-glycaemic-load dietary intervention for the management of glycaemia and serum lipids among women with gestational diabetes: A randomized control trial. Public Health Nutr..

[21] Wan C.S., Nankervis A., Teede H., Aroni R. (2019). Dietary intervention strategies for ethnic Chinese women with gestational diabetes mellitus: A systematic review and meta-analysis. Nutr. Diet..

[22] Mustafa S.T., Harding J.E., Wall C.R., Crowther C.A. (2022). Adherence to Clinical Practice Guideline Recommendations in Women with Gestational Diabetes and Associations with Maternal and Infant Health-A Cohort Study. Nutrients.

[23] Vestgaard M., Christensen A.S., Viggers L., Lauszus F.F. (2017). Birth weight and its relation with medical nutrition therapy in gestational diabetes. Arch. Gynecol. Obstet..

[24] Guo M., Li Y., Liu D., Zhang L., Zhang W. (2015). Effects of Individualized Nutrition Intervention on Pregnant Women with Gestational Diabetes Mellitus. J. Clin. Res..

[25] Michie S., van Stralen M.M., West R. (2011). The behaviour change wheel: A new method for characterising and designing behaviour change interventions. Implement. Sci..

[26] Gould G.S., Bar-Zeev Y., Bovill M., Atkins L., Gruppetta M., Clarke M.J., Bonevski B. (2017). Designing an implementation intervention with the Behaviour Change Wheel for health provider smoking cessation care for Australian Indigenous pregnant women. Implement. Sci..

[27] Li F., Shen H., Qin K., Wang M., Song W., Fan X. (2018). Application of healtheducation modelbased on BCW in patients with cancer pain. Chin. J. Cancer Prev. Treat..

[28] Metzger B.E., Gabbe S.G., Persson B., Buchanan T.A., Catalano P.A., Damm P., Dyer A.R., Leiva A., Hod M., Kitzmiler J.L. (2010). International association of diabetes and pregnancy study groups recommendations on the diagnosis and classification of hyperglycemia in pregnancy. Diabetes Care.

[29] Hertzog M.A. (2008). Considerations in determining sample size for pilot studies. Res. Nurs. Health.

[30] Reader D., Splett P., Gunderson E.P. (2006). Impact of gestational diabetes mellitus nutrition practice guidelines implemented by registered dietitians on pregnancy outcomes. J. Am. Diet. Assoc..

[31] Duarte-Gardea M.O., Gonzales-Pacheco D.M., Reader D.M., Thomas A.M., Wang S.R., Gregory R.P., Piemonte T.A., Thompson K.L., Moloney L. (2018). Academy of Nutrition and Dietetics Gestational Diabetes Evidence-Based Nutrition Practice Guideline. J. Acad. Nutr. Diet..

[32] Dietary Guide for Patients with Gestational Diabetes Mellitus (GDM): WS/T 601—2018. http://www.nhc.gov.cn/wjw/yingyang/201805/aa6da5f40b1947cc975e30327551fae9.shtml.

[33] Bijl J.V., Poelgeest-Eeltink A.V., Shortridge-Baggett L. (1999). The psychometric properties of the diabetes management self-efficacy scale for patients with type 2 diabetes mellitus. J. Adv. Nurs..

[34] Vivienne Wu S.F., Courtney M., Edwards H., McDowell J., Shortridge-Baggett L.M., Chang P.J. (2008). Development and validation of the Chinese version of the Diabetes Management Self-efficacy Scale. Int. J. Nurs. Stud..

