# Supplementary material for: Effect of Dietary Intervention Designed with Behavior Change Wheel on Compliance with Dietary Control in Women with Gestational Diabetes Mellitus: Study Protocol for a Randomized Controlled Trial
Source: Int J Environ Res Public Health. 2022 Aug 28;19(17):10726. doi: 10.3390/ijerph191710726 (PMC9518101; doi:10.3390/ijerph191710726)
Supplement: Supplementary file 1 [file ijerph-19-10726-s001.zip › ijerph-1846916-supplementary.pdf]

Supplementary File S1:

Equation (S1). The calculation of pre-pregnancy body mass index

$$\text{Pre- pregnancy BMI} = \frac{\text{Weight(kg)}}{\text{Height(m)}^2}$$

Equation (S2). The calculation of daily energy requirements

(1) The calculation of standard weight

Standard weight (kg)=Height(cm)-105

(2) The calculation of total energy requirement

Total energy (kcal/per day)=Standard weight(kg)×Energy amount (per kg/per day)+300kcal (second trimester)/450kcal (third trimester)

Equation (S3). The calculation of number of food exchange serving

$$\text{Number of food exchange serving} = \frac{\text{Total Energy(kcal)}}{90}$$

Supplementary File S2

Table S1. The classification of body type in women with GDM according to pre-pregnancy BMI.

Pre-pregnancy BMI	Body type

BMI<18.5 (kg/m2)	Thin
18.5≤BMI≤24.9 (kg/m2)	Normal
25.0≤BMI<30.0 (kg/m2)	Overweight
BMI≥30.0 (kg/m2)	Obesity

Table S2. Daily energy requirement based on labor intensity of Chinese adults.

**Labor Intensity**	**Examples of Job Categories**	**Daily Energy Requirements (Standard Weight per kg)**		
		**Thin**	**Normal**	**Overweight or Obesity**
Rest on bed	——	20-25	15-20	15
Light physical labor	Office staff, teachers, and housewives.	35	30	20-25
Moderate physical	Students, drivers, doctors, and farmers	40	35	30

labor	(general farm work).			
Heavy physical labor	Builders, porters, and athletes.	45	40	35

Supplementary File S3:

1. “How many days a week do you eat according to the principle of "breakfast, lunch and dinner, and 2-4 extra meals?”;
2. “How many days a week do you match the staple food according to the principle that the ratio of coarse grain to fine grain is 1: 2 or 1: 4?”;
3. “How many days a week do you subtract the corresponding staple food when eating starchy foods (for example, 100 grams of potatoes can be exchanged with 25 grams of rice, millet and sorghum rice)?”;
4. “How many days a week do you follow the food exchange strategy as recommended by the medical staff?”;
5. “How many days a week do you refer to the GL value of food when choosing foods?”
